# Quantum Implementation of the SAND Algorithm and Its Quantum Resource Estimation for Brute-Force Attack

**DOI:** 10.3390/e26030216

**Published:** 2024-02-29

**Authors:** Hongyu Wu, Xiaoning Feng, Jiale Zhang

**Affiliations:** 1 College of Computer Science and Technology, Harbin Engineering University, Harbin 150001, China; b221060008@hrbeu.edu.cn; 2 College of Computer Science and Technology, Jilin University, Changchun 130012, China; jlzhang22@mails.jlu.edu.cn

**Keywords:** grover algorithm, brute-force attack, SAND algorithm, lightweight block cipher

## Abstract

The SAND algorithm is a family of lightweight AND-RX block ciphers released by DCC in 2022. Our research focuses on assessing the security of SAND with a quantum computation model. This paper presents the first quantum implementation of SAND (including two versions of SAND, SAND-64 and SAND-128). Considering the depth-times-width metric, the quantum circuit implementation of the SAND algorithm demonstrates a relatively lower consumption of quantum resources than that of the quantum implementations of existing lightweight algorithms. A generalized Grover-based brute-force attack framework was implemented and employed to perform attacks on two versions of the SAND algorithm. This framework utilized the g-database algorithm, which considered different plaintext–ciphertext pairs in a unified manner, reducing quantum resource consumption. Our findings indicate that the SAND-128 algorithm achieved the NIST security level I, while the SAND-64 algorithm fell short of meeting the requirements of security level I.

## 1. Introduction

The advent of quantum computers and quantum algorithms has dramatically changed the cryptography community. The quantum computation model is expected to bring about profound alterations in the current landscape of cryptanalysis [1,2]. Due to the emergence of two pioneering quantum algorithms—the Shor algorithm [3] and Grover algorithm [4]—the current classical cryptosystem is under threat. An efficient quantum algorithm for solving the large integer factorization problem was provided by the Shor algorithm, which can break most currently used public-key systems, such as RSA cryptosystems and elliptic curve cryptography. As a high-performance quantum search algorithm, the Grover algorithm can reduce the cost of a brute-force attack on a *k*-bit key from $2^{k}$ to $2^{k/2}$.

The quantum implementation of classical encryption algorithms and the evaluation of quantum resources are of great significance. First, quantum implementations of classical algorithms are beneficial for evaluating the security strength of ciphers in quantum computation models. In the post-quantum era, the National Institute of Standards and Technology (NIST) has proposed the use of the cost of brute-force attacks based on a Grover search as an indicator of the security strength of a cryptographic system [5]. The process of performing a brute-force attack using the Grover algorithm requires efficient quantum implementations of classical encryption schemes. Second, the quantum implementation of classical algorithms is conducive to exploring the security issues of the quantum Internet. One of the security measures for linking a single quantum computer to the quantum internet is a quantum implementation of a classical encryption algorithm [6,7]. To sum up the above two points, it is necessary to design a quantum circuit of classical encryption algorithms.

The T-depth and qubits are frequently considered metrics in quantum resource consumption [8,9]. The T-depth of quantum circuits is a critical metric for circuit reliability, and its reduction leads to effective minimization of noise accumulation, thereby resulting in improvements of the fault tolerance of the circuits [10,11]. Additionally, the number of qubits plays a crucial role in the execution time, error rate, and computing power of a quantum system [12,13]. The consideration of the ’depth-times-width’ metric, wherein the depth is defined as the T-depth and the width is equivalent to the number of qubits, captures the comprehensive resource requirements of quantum circuits and offers a more holistic understanding of a circuit’s resource demands.

Due to the high performance and popularity of the Advanced Encryption Standard (AES) algorithm [14], the quantum implementation and quantum resource evaluation of the AES algorithm have received sufficient attention [15,16,17,18,19,20]. In the post-quantum cryptography (PQC) standardization process, the NIST defined security categories by evaluating the difficulty of conducting a brute-force key attack. For a meaningful definition of the security categories, NIST derives security I∼V from the gate and depth cost estimates for a brute-force attack on the AES algorithm by Jaques et al. [17].

New lightweight symmetric algorithms are rapidly developing in Internet of Things (IoT) networks [21]. With the rise of the IoT, lightweight symmetric encryption algorithms are rapidly gaining prominence [22]. Quantum implementation and quantum evaluation of lightweight cryptographic encryption algorithms are currently being carried out with great enthusiasm. Lin et al. and Zou et al. implemented the quantum version of the Chinese commercial cipher standard, i.e., SM4 [23,24]. Considering metrics such as the depth-times-width metric, the security of the SM4 algorithm against quantum brute-force attacks was weaker than that of the AES-128 algorithm. Bathe et al. analyzed the ChaCha algorithm, which is commonly used in embedded devices, and evaluated the quantum resources required by the Grover algorithm for ChaCha [25]. Jang et al. simultaneously considered various lightweight algorithms such as HIGHT, CHAM, LEA, and the NSA-developed SPECK cipher [26]. The results indicated that CHAM required the least quantum resources, implying that CHAM’s block cipher was the most vulnerable to attacks [26,27]. The immense enthusiasm for this field motivates us to seek a lightweight cryptographic algorithm with lower quantum resource requirements and to attain its NIST security rating.

The S-box-based and AND-RX based structures (SAND) algorithm, which has emerged in recent years, has shown great potential among the new lightweight symmetric algorithms [28]. The SAND algorithm has advantages, including a simplified key schedule and competitive software performance. The SAND algorithm is often applied to resource-constrained devices to provide data protection and communication security. Furthermore, the SAND algorithm can undergo a comprehensive security analysis under classical conditions, such as differential and linear attacks and single-key and related-key scenarios. However, it is worth noting that there has been no comprehensive security analysis of SAND in the context of the quantum computation model.

**Our Contributions.** The main purpose of our research is to determine the security of the SAND algorithm within the quantum computation model—specifically, a brute-force attack based on the Grover algorithm. Our contributions are two-fold and can be described as follows:

1. *The quantum implementation of the SAND algorithm.* We present the first quantum circuit implementation of the SAND cryptographic algorithm by optimizing a combination of Toffoli, CNOT, X, and SWAP gates. The process begins with the design of various small components of the SAND algorithm, which are then used as a foundation for designing the round function and key schedule. Subsequently, a quantum implementation of the SAND algorithm is constructed. A comparison is provided, contrasting its resource consumption with that of other recently implemented lightweight cryptographic quantum circuits. The analysis, which includes the depth-times-width indicator, reveals that the resource consumption of quantum circuits for the SAND cryptographic algorithm is relatively low.
2. *Quantum brute-force attack on the SAND algorithm.* We created a generalized brute-force attack framework by introducing the g-database algorithm, which took different plaintext–ciphertext pairs into account in a unified manner and was able to reduce the consumption of quantum resources (it could reduce the number of qubits and gate consumption to approximately 1/r of those of the original quantum circuit; *r* is the number of different plaintext–ciphertext pairs). Based on the quantum implementation of quantum SAND, the quantum resource cost was estimated within the generalized brute-force attack framework. It was revealed that the SAND-128 algorithm achieved the NIST security level I, while the SAND-64 algorithm fell short of meeting the requirements of security level I.

**Organization.** Section 2 introduces the symbols used in this paper and briefly introduces the Grover algorithm and SAND algorithm. Section 3 provides the quantum implementation of the SAND algorithm. In Section 4, we establish a generalized brute-force attack framework based on the Grover algorithm within the quantum computation model and apply this framework to the SAND algorithm. Section 5 evaluates and compares the quantum resource consumption of the brute-force attack. Section 6 summarizes the work of this study.

## 2. Preliminaries

### 2.1. Symbol Description

In this section, we introduce the various symbols used in SAND and the quantum computation operations that are commonly used in this paper.

The main symbols in this paper are shown in Table 1. The following 4×n/4 two-dimensional matrix is used to represent the variable *x* in this paper:

(1) $$x=\left[\begin{array}{cccc} x_{n-1} & \cdots & x_{7} & x_{3}\\ x_{n-2} & \cdots & x_{6} & x_{2}\\ x_{n-3} & \cdots & x_{5} & x_{1}\\ x_{n-4} & \cdots & x_{4} & x_{0} \end{array}\right]=\left[\begin{array}{c} x\{3\}\\ x\{2\}\\ x\{1\}\\ x\{0\} \end{array}\right]=\left[\begin{array}{c} x\left[\frac{n}{4}-1\right]\;\cdots \;x\left[1\right]\;x\left[0\right] \end{array}\right]$$

**Figure 1:**
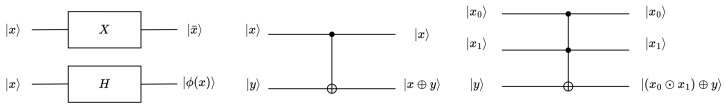
Quantum circuit gates (X gate, CNOT gate, Toffoli gate).

**Figure 2:**
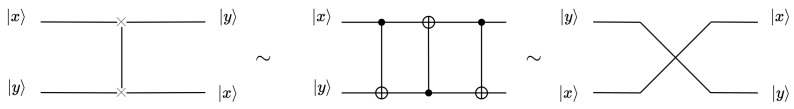
Quantum circuit gate (SWAP gate).

Quantum computers use quantum gates to operate on qubits. Figure 1 shows the X gate, H gate, CNOT gate, and Toffoli gate. The X gate (also known as the NOT gate) inverts the input qubit. The H gate creates a superposition of states. Suppose that the state of the input qubit is |x〉, where *x* can be 0 or 1; the output qubit is $|\phi \left(x\right)\rangle =\frac{1}{\sqrt{2}}(|0\rangle +{\left(-1\right)}^{x}\left|1\rangle \right)$ through the H gate. The two input states of the CNOT gate are called the control qubit ∣x〉 and the target qubit ∣y〉. After the CNOT gate, the control qubit remains unchanged, and the target qubit becomes ∣x⊕y〉. The Toffoli gate, which can have multiple control qubits, is used in this study with only two control qubits. After passing through the Toffoli gate, the control bits remain unchanged, and the target qubit becomes $|(x_{0}⊙x_{1})⊕y\rangle$. The SWAP gate can be constructed using three CNOT gates, as illustrated in Figure 2, and the three representations in the figure are equivalent.

### 2.2. Grover Algorithm

Given the database f(x) with *N* entries, the primary key *w* that makes a data record of f(w)=1 is found. The Grover algorithm is a database search algorithm that mainly provides quadratic acceleration about the above search problem of an unstructured database f(x). The main procedures of the Grover algorithm are as follows:

1. An equal-weight superposition state $H^{⊗n}{|0\rangle }^{⊗n}=\frac{1}{2^{n/2}}\sum_{x=0}^{2^{n}-1}|x\rangle$ is prepared.
2. The Grover iteration is repeated κ times, $\kappa \approx \frac{\pi }{4}\sqrt{N}$:
  (a) The $U_{w}$ operator is applied, where $U_{w}=I-2|$w〉〈w∣.
  (b) The Grover diffusion operator $U_{s}$ is applied; $U_{s}=2|$s〉〈s∣−I.
3. The result is measured as f(w) with a very high probability.

**Figure 3:**
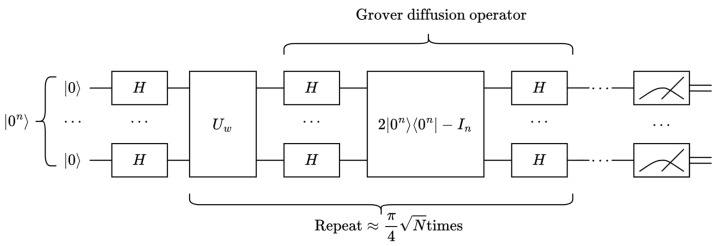
Quantum circuit diagram of the Grover algorithm.

For sufficiently large *N*, there is $sin\theta =\frac{1}{\sqrt{N}}\approx \theta$. So, the number of iterations κ can also be written as $\frac{\pi }{4\theta }$. The quantum circuit diagram of the Grover algorithm is shown in Figure 3.

### 2.3. SAND Algorithm

SAND is a family of AND-recursive exchange (AND-RX) block ciphers with the Feistel structure, and it includes SAND-64 and SAND-128 versions [28]. They both support 2n plaintext with a 128-bit key. The basic parameters of SAND-64 and SAND-128 are listed in Table 2.

**Figure 4:**
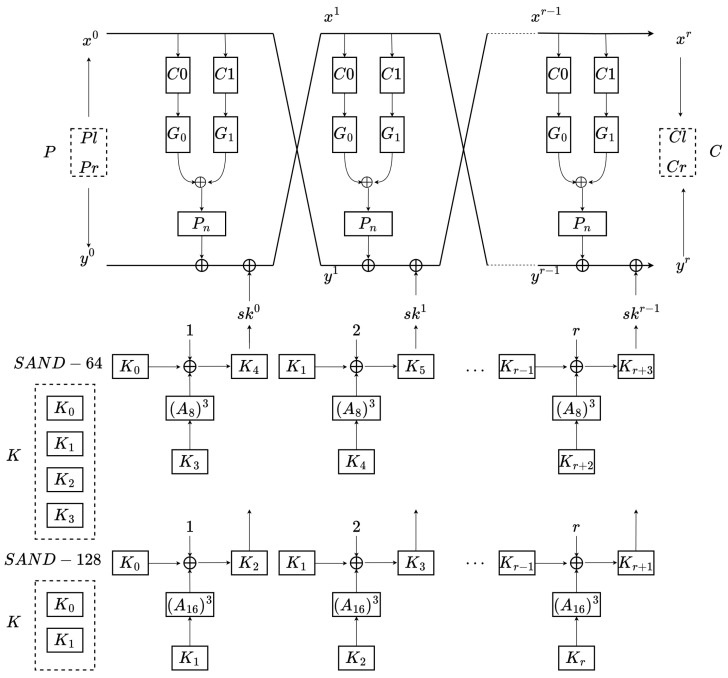
SAND encryption algorithm.

The overall structure of SAND is shown in Figure 4. The input plaintext P=(Pl,Pr) is encrypted by the key $K=K^{3}\|K^{2}\|K^{1}\|K^{0}$ in SAND-64 or the key $K=K^{1}\|K^{0}$ in SAND-128. The final output is the ciphertext C=(Cl,Cr). The classical SAND algorithm is mainly divided into a round function and a key schedule. The round functions of SAND-64 and SAND-128 can be expressed as follows:

(2) $$\left(x^{r+1},y^{r+1}\right)=F_{sk^{r}}\left(x^{r},y^{r}\right)=\left(P_{n}\left(G_{0}\left(C0(x^{r})\right)⊕G_{1}\left(C1(x^{r})\right)\right)⊕y^{r}⊕sk^{r},x^{r}\right)$$

The tuples of (C0,C1) are the rotation components, and  $C0\left(x^{r}\right)=x^{r}{⋘}_{n/4}\alpha ,\;$$C1\left(x^{r}\right)=x^{r}{⋘}_{n/4}\beta$. The tuple of the rotation constants (α,β) is fixed to (0,1) for all versions of SAND.

$G_{0}$ and $G_{1}$ are nonlinear functions. Their input is x{3}||x{2}||x{1}||x{0}, and their output is y{3}||y{2}||y{1}||y{0}. For $G_{0}$,

(3) $$\begin{array}{c} y\{0\}=x\{3\}⊙x\{2\}⊕x\{0\}\\ y\{3\}=y\{0\}⊙x\{1\}⊕x\{3\}\\ y\{2\}=x\{2\}\\ y\{1\}=x\{1\} \end{array}$$

As for the function G1, the output is calculated as follows:

(4) $$\begin{array}{c} y\{2\}=x\{3\}⊙x\{1\}⊕x\{2\}\\ y\{1\}=y\{2\}⊙x\{0\}⊕x\{1\}\\ y\{3\}=x\{3\}\\ y\{0\}=x\{0\} \end{array}$$

The $P_{n}$ box is rearranged for a variable *x* on 4 rows. For the *i*-th row input $x\left\{i\right\}=\left(x_{\frac{n}{4}\cdot i+\frac{n}{4}-1},\ldots ,x_{\frac{n}{4}\cdot i+1},x_{\frac{n}{4}\cdot i}\right)$, the element of the *i*-th output row y{i} is defined as follows:

(5) $$y_{\frac{n}{4}\cdot i+p_{\frac{n}{4}}\left(j\right)}=x_{\frac{n}{4}\cdot i+j}\;\mathrm{for}\;0\leq j<\frac{n}{4},0\leq i<4$$

where $p_{8}$ and $p_{16}$ are used in SAND-64($P_{32}$) and SAND-128($P_{64}$). The permutations of $p_{8}$ and $p_{16}$ are listed in Table 3 and Table 4, respectively.

The initial key bits of SAND-64 and SAND-128 are both 128 bits. The *r*-th round subkey $sk^{r}\left(0\leq r\leq R\right)$ is loaded from *K*. SAND-64 treats the 128-bit key as $K=K^{3}\|K^{2}\left\|K^{1}\right\|K^{0}$ (four 32-bit words). SAND-128 treats the 128-bit key as $K^{1}\|K^{0}$ (two 64-bit words). The equations for the key schedule are:

(6) $$\begin{array}{cc} \; & K^{i+4}={\left(A_{8}\right)}^{3}\left(K^{i+3}\right)⊕K^{i}⊕\left(i+1\right)\;\;\;\;\mathrm{for}\;\mathrm{SAND}-64\\ \; & K^{j+2}={\left(A_{16}\right)}^{3}\left(K^{j+1}\right)⊕K^{j}⊕\left(j+1\right)\;\;\;\;\mathrm{for}\;\mathrm{SAND}-128 \end{array}$$

where (i+1,j+1) is the round constant (RC), and  0≤i<R−4,0≤j<R−2.

$A_{8}$ and $A_{16}$ are nibble-oriented functions. The input of $A_{8}$ is $X\left[7\right]\left\|\cdots X\left[1\right]\right\|X\left[0\right]$, and the output of $A_{8}$ is $X^{\prime }\left[7\right]\left\|\cdots X^{\prime }\left[1\right]\right\|X^{\prime }\left[0\right]$:

$$\left(X\left[7\right]⋘t_{1}\right)⊕X\left[0\right]\|X\left[7\right]⊕\left(X\left[7\right]\ll t_{0}\right)\|X\left[6\right]\|X\left[5\right]\|X\left[4\right]\|X\left[3\right]\|X\left[2\right]\|X\left[1\right]$$

where $t_{0}$ and $t_{1}$ are set to 3 and 1, respectively.

The input of $A_{16}$ is $X\left[15\right]\left\|\cdots X\left[1\right]\right\|X\left[0\right]$, and the output of $A_{16}$ is $X^{\prime }\left[15\right]\left\|\cdots X^{\prime }\left[1\right]\right\|X^{\prime }\left[0\right]$:

$$\left(X\left[15\right]⋘t_{1}\right)⊕X\left[0\right]||X\left[15\right]⊕\left(X\left[15\right]\ll t_{0}\right)\|X\left[14\right]\|X\left[13\right]\|\cdots \|X\left[2\right]\|X\left[1\right]$$

where the same settings for $t_{0}$ and $t_{1}$ are used as in $A_{8}$, with $t_{0}$ being 3 and $t_{1}$ being 1.

## 3. Quantum Implementation of SAND

### 3.1. Quantum Implementation of Small Components of SAND

In this subsection, the quantum implementation of the the rotating movement operation, shift movement operation, nonlinear functions, $P_{n}$ box, $A_{8}$, and $A_{16}$ is introduced. These small components are necessary for implementing the key schedule and round function of the SAND algorithm.

Algorithm 1 implements |x{i}⋘t〉. A left-rotating *t*-qubit movement operation consists of three reverse operators. The reverse operator in Algorithm 2 is written back to the same qubit. The reverse operator whose input is $|x_{0}x_{1}\ldots x_{n-1}x_{n}\rangle$ requires ⌊n/2⌋ SWAP operations, and the order of the SWAP operations can be changed. A rotating *t*-qubit movement operation is performed on each of the rows, and this is implemented in series to form $|x{⋘}_{n/4}t\rangle$.
**Algorithm 1** Rotating movement operation**Input:**$|x_{0}\ldots x_{n}\rangle$, *t* **Output:**$|x_{t}\ldots x_{n}x_{0}\ldots x_{t-1}\rangle$ 1: Reverse($|x_{0},\ldots ,x_{t-1}\rangle$) 2: Reverse($|x_{t},\ldots ,x_{n}\rangle$) 3: Reverse($|x_{0}x_{1}\ldots x_{n}\rangle$)

**Algorithm 2** Reverse  operator**Input:**$|x_{0}x_{1}\ldots x_{n-1}x_{n}\rangle$ **Output:**$|x_{n}x_{n-1}\ldots x_{1}x_{0}\rangle$ 1: SWAP($x_{0},x_{n}$) 2: … 3: SWAP($x_{n/2},x_{n/2+1}$)

An *n*-qubit to *n*-qubit shift movement operation requires n−1 SWAP gates, i.e., SWAP($x_{0},x_{1}$), SWAP($x_{1},x_{2}$), …, SWAP($x_{n-1},x_{n}$). The reset operation, as described for the INIT gate in Section 3.2, is applied to the last qubit.

**Figure 5:**
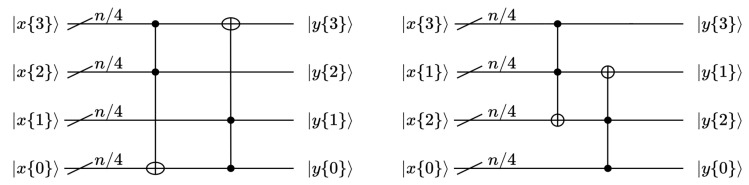
Quantum circuit diagram of $G_{0},G_{1}$.

According to Equations (3) and (4), the Toffoli gate is used to implement $G_{0}$ and $G_{1}$, as shown in Figure 5. Naturally, the object of the AND operation in the SAND algorithm is mapped to the control circuit of the Toffoli gate. The object of the XOR operation in the SAND algorithm is mapped to the target circuit.

$p_{8}$ and $p_{16}$ are obtained through an optimization design, as shown in Table 5. We used 7 SWAP gates and 14 SWAP gates, respectively, to implement the $p_{8}$ and $p_{16}$ gates. The $P_{n}$ boxes of SAND-64 and SAND-128 were implemented with $p_{8}$ or $p_{16}$, respectively.

**Figure 6:**
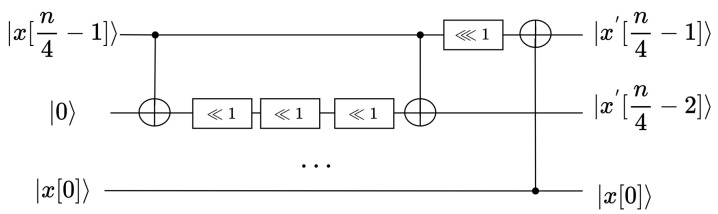
The quantum circuit diagram of $A_{8},A_{16}$.

The implementations of the rotating movement operation and shift movement operation were embedded into the implementations of $A_{8}$ and $A_{16}$, with the only difference being in the input qubits. The implementation of $A_{8},\;A_{16}$ is depicted in Figure 6. Although an additional nibble qubit was added, $A_{8}$ and $A_{16}$ did not add a large number of qubits due to qubit reuse technology [29,30]. The colons in the figure are all operations that modify the qubit index, which is not counted as a resource in most cases.

### 3.2. Quantum Implementation of SAND’s Round Function

**Figure 7:**
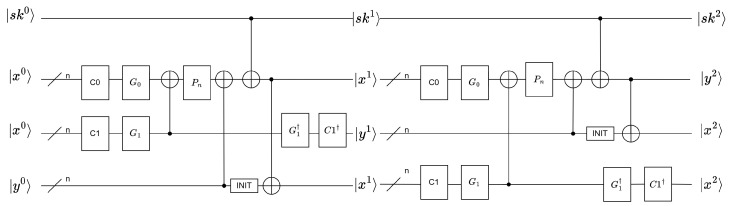
The quantum circuit diagram of the round function $R_{1}$$R_{2}$ of two-round SAND.

The key of each round was set to $sk^{i}$, 0≤i≤R−1, temporarily ignoring the key schedule part. Auxiliary qubits were introduced to hold a copy of the state of $x^{r}$. An INIT gate was concurrently required to restore an unknown state to the |00…0〉 state. The implementation of the INIT gate involved using the X gate to modify the measured unknown state to obtain |00…0〉. Subsequently, |00…0〉 was incorporated into the original quantum circuit (The INIT gate corresponds to Qiskit’s Reset() operation, ensuring that the qubit is reset to a known initial state. In this circuit, it is used to reset qubits to their ground state |0〉). Moreover, the inverse transformation of C1 and $G_{1}$ was used to obtain the intermediate state $y^{i}$, 1≤i≤R. As C1 and $G_{1}$ only used basic circuit gates, the inverse transformation of C1 and $G_{1}$ needed to exist. The quantum circuit diagram of the two-round SAND round function is shown in Figure 7.

The round function of each round was transformed as follows:

$$\begin{array}{cc} x^{i+1} & =P_{n}\left(G_{0}\left(C0\left(x^{i}\right)\right)⊕G_{1}\left(C1\left(x^{i}\right)\right)\right)⊕y^{i}⊕sk^{i}\\ y^{i+1} & =C1^{†}\left(G_{1}^{†}\left(G_{1}\left(C1\left(x^{i}\right)\right)\right)\right)\\ x^{i+1} & =CNOT(x^{i+1},INIT\left(y^{i}\right)) \end{array}$$

### 3.3. Quantum Implementation of the Key Schedule

**Figure 8:**
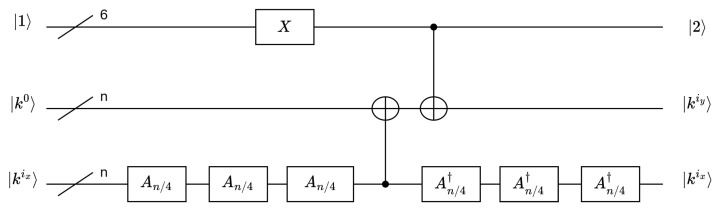
Quantum circuit diagrams of the key schedules of SAND-64 and SAND-128.

According to Equation (6) of the key schedule and the output forms of $A_{8}$ and $A_{16}$, the quantum circuit diagrams for the first round of the key schedule for SAND-64 and SAND-128 are shown in Figure 8. In this diagram, $\left(i_{x},i_{y}\right)$ are represented as (3,4) for SAND-64 and (1,2) for SAND-128. The fundamental concept was the incorporation of $k^{i_{min}}$ as the update component in the key schedule for each round.

To achieve RC, 6 qubits were necessary. A 6-bit auxiliary bit was sufficient for both SAND versions because of $2^{6}>$ 54. The initial state of the auxiliary qubits was |1〉. These qubits went through X gates to facilitate an increment in the quantum state (+ 1 operations) in each round, resulting in RC through R + 1 operations. Specifically, SAND-64 required 94 X gates, while SAND-128 necessitated 104 X gates in the 6-qubit configuration.

### 3.4. Quantum Implementation of SAND

**Figure 9:**
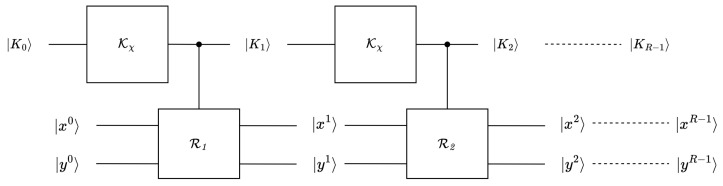
Quantum circuit diagram of SAND.

The round function $R_{i}$ and key schedule Kχ of SAND were combined as a subgate in SAND as a whole. We implemented SAND as a reversible circuit because reversibility was necessary for the cipher to be useful as a subroutine in the Grover search. With the circuits developed for the round function and key schedule, the circuit for full-round SAND could be constructed. The initial state was designated as $\left(K_{0},x^{0},y^{0}\right)$, and the state propagated through *i* rounds as $\left(K_{1},x^{1},y^{1}\right),\left(K_{2},x^{2},y^{2}\right),\ldots ,\left(K_{i},x^{i},y^{i}\right),0\leq i<R-1$. The quantum circuit diagram of the implementation is shown in Figure 9.

## 4. Generalized Brute-Force Attack Framework Based on the Grover Algorithm

In this section, we describe the application of the Grover algorithm to brute-force attacks, and we establish a generalized brute-force attack framework for SAND under known plaintext attacks. SA represents the entire SAND encryption algorithm; let $\left(P_{i},C_{i}\right)$ be multiple sets of plaintext–ciphertext pairs. Each pair of plaintext units $P_{i}$ is assigned a key *K* in SA, which is encrypted as $C_{i}$, namely, $C_{i}=SA\left(P_{i},K\right)$. When the number of plaintext–ciphertext pairs is only 1, there are multiple keys *K* satisfying the SAND encryption algorithm, namely,

$$C_{1}=SA\left(P_{1},K_{0}\right);\;C_{1}=SA\left(P_{1},K_{1}\right);\ldots$$

So, in practice, brute-force attacks consider multiple plaintext–ciphertext pairs. Let $r=2^{\rho }$ plaintext–ciphertext pairs be sufficient to successfully extract a unique *K*. Given a key that has a number of possible entries $N=2^{n}$, in order to find a unique key *K*, the $U_{w}$ operator (in the Grover algorithm) is defined as

(7) $$\begin{array}{cc} \; & f\left(K\right)=1\;\mathrm{iff}\;C_{i}=SA\left(P_{i},K\right),1\leq i\;\leq 2^{\rho };0,\mathrm{otherwise}.\\ \; & U_{w}|K\rangle ={\left(-1\right)}^{f(K)}|K\rangle \end{array}$$

Brute-force attacks usually require 2∼4 plaintext–ciphertext pairs [15,17,23]. *r* can be determined through the key size *k* and branch size *n*, i.e.,  r≥⌈k/n⌉ [17]. So, *r* must be at least (2,4) for SAND-128 and SAND-64. In the $U_{w}$ operator of the Grover algorithm, 2rSA instances are required for parallel testing of a brute-force attack. Next, we detail the brute-force attack framework.

The main purpose of the g-database algorithm is to obtain corresponding superposition states of ciphertexts with plaintexts. In order to obtain the superposition of ciphertexts, we incorporate the g-database algorithm from [31] into the brute-force attack framework. Querying the entire codebook in the g-database algorithm appears redundant for the stated purposes, as the entire codebook is not required to determine a key. We made appropriate modifications to the g-database algorithm and embedded it into our circuit. For the specific g-database algorithm, see Algorithm 3.
**Algorithm 3** Modified g-database algorithm.**Input:**${|0\rangle }^{⊗N}{|0\rangle }^{⊗N}$, classical query access to g **Output:** The g-database
$$|r_{g}\rangle =|x\rangle |g\left(x\right)\rangle$$ 1: H-gate operations are performed on ρ qubits (not necessarily continuous) for the front ${|0\rangle }^{⊗N}$.
$${|x\rangle |0\rangle }^{⊗N}$$ 2: For each $x\in {\left\{0,1\right\}}^{N}$, $2^{\rho }$ classical queries g(x) are performed, and then the g(x) unitary operator is applied in the second register. 3: **Return**
|x〉|g(x)〉▷ Return the g-database $|r_{g}\rangle$

The foundational logic of the g-database algorithm involves classical querying of all instances of g(x) and the subsequent application of the resulting g(x) unitary operator to the state |x〉|0〉, effectively establishing g(x) on the second register. Our main change is as follows: The plaintext state is specified in the first step (one of the forms created is $|x\rangle =\underset{\underset{\rho \;\;|H\rangle \;\;states}{⏟}}{\ldots |H\rangle \ldots |0\rangle \ldots }$). We only need to accurately classically query g(x)$2^{\rho }$ times, and then we can apply the resulting unitary g(x) operator to |x〉|0〉, which forms |g(x)〉 on the second register. The usage of quantum resources is reduced by transforming the original querying of the SA quantum circuit into classical queries.

**Figure 10:**
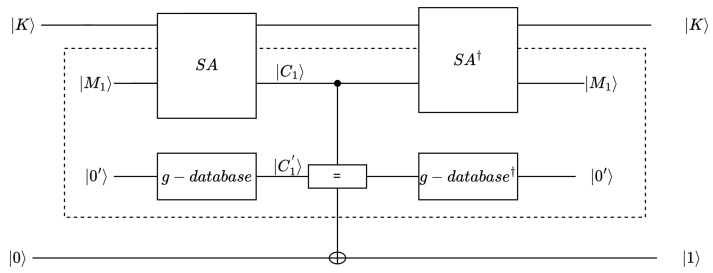
$U_{w}$ operator of the Grover algorithm for brute-force attacks on SAND. The EM model [35] and the form of the g−database unitary matrix are used to represent $U_{w}$.

Figure 10 shows the important $U_{w}$ operator in the Grover algorithm. For the initial state $|0^{\prime }\rangle$, the ciphertext superposition state $|C_{1}^{\prime }\rangle$ is obtained using the g-database algorithm. Then, the state $|C_{1}^{\prime }\rangle$ is compared with $|C_{1}\rangle$, which is obtained through the SA circuit. If the ciphertext states are consistent, the target quantum qubit will be flipped (The state distinction principle [32] is a technique widely used in quantum signature schemes [33,34]. Quantum superposition states can be compared, and it can be determined whether the key is correct.). Specifically, if $|C_{1}^{\prime }\rangle =|C_{1}\rangle$, the quantum comparator outputs 1, and the output will be flipped; otherwise, the quantum comparator outputs 0 or the output is unchanged (the principle of state distinguishing asserts that if two unknown states are identical, measuring a result of ’0’ becomes impossible).

Regarding the Grover algorithm, it is commonly stated that it necessitates approximately $\frac{\pi }{4}\sqrt{N}$ iterations. This does not mean that queries of the g-database algorithm (which corresponds to the classical SAND algorithm) need to be performed in every iteration. This is because the g-database algorithm can be consolidated into a single-g-database unitary matrix. We can store the queried data in a data table and generate a unitary matrix based on this data table for each iteration. This framework can reduce the number of qubits and gate consumption to approximately 1/r of the original circuit.

This brute-force attack framework can be seen as a generalization of a previously proposed brute-force attack framework. Specifically, when the g-database unitary matrix transforms into the r−1SA unitary matrix, our framework is identical to the previous attack framework. Note that the quantum resource consumption of the g-database unitary matrix is less than that of the SA circuit. This conclusion is based on an intuitive assumption that the greater the power of a unitary matrix, the more quantum resources it requires. In terms of the power of unitary matrices, the SA matrix can encrypt the plaintext state $x\in {\left\{0,1\right\}}^{n}$ into a ciphertext state for all inputs. On the other hand, the g-database unitary matrix can only encrypt a subset of the plaintext states into ciphertext states.

In the Q1 model, the attacker is allowed to make classical queries to the encryption oracle but has access to a quantum computer for making offline computations. In the Q2 model, besides having access to a quantum computer, the attacker is allowed to make superposition queries to the oracle. The way of realizing cryptographic protocols (the SAND algorithm) by using quantum resources so that they can be quantum superposition queries with the Grover algorithm belongs to the Q2 model.

## 5. Attack Analysis and Evaluation

Quantum circuit depth is determined by the number of quantum gates in a column, which consists of basic or physical gates (such as Clifford gates and T gates) or combinational gates (such as Toffoli gates). Researchers have explored techniques for decomposing Toffoli gates [36,37]. Specifically, their goal was to optimize the arrangement of T gates and Clifford gates, resulting in maximum T-gate parallelism to reduce the depth of T gates.

We performed the decomposition of Toffoli gates to the Clifford+T level. A Toffoli gate was decomposed into 7 T gates and 8 Clifford gates (6 CNOT gates and 2 H gates), with the T-depth being 4 and the full depth (total depth) being 8 according to the method presented in [37]. Based on [37], the depth of a series of circuits was optimized using Qiskit, ensuring that it was less than or equal to the sum of the individual depths and T-depths (Qiskit allows us to automatically compute circuit depth by moving gates around through a circuit if the qubits that they act on were previously idle. This means that the depth of two circuits applied in series may be less than the sum of the individual depths of each circuit.). The optimized $G_{0}$ and $G_{1}$ of SAND-64 are shown in Appendix A.

We first calculated the quantum resources required for each round of the SAND algorithm. As illustrated in Figure 2, the SWAP gate was decomposed into a sequence of three CNOT gates for the purpose of resource estimation. This approach differed from the methodologies adopted in [17,24,25,27], where SWAP gates were not typically accounted for as separate resources. While this strategy did indeed increase the overall depth and the quantum gate count of the circuit, it significantly enhanced the transparency and accuracy of quantum resource estimation. The implementation of the INIT gate involved the μ/2 gate, where μ denotes the number of qubits on which the INIT gate acted.

Through the quantum resource consumption of each component shown in Table 6, the quantum resource consumption of one round of the SAND algorithm could be obtained. The round function required 3n qubits, and the key schedule needed k+10 qubits (four qubits to implement $A_{n/4}$ and six qubits to implement the round constant). Table 7 illustrates the quantum resources consumed by each round of the SAND algorithm.

The quantum resources for each round of the SAND algorithm were multiplied by *R* to yield an estimation of the quantum resources consumed for a single encryption by the SAND algorithm. The full depth of the SAND algorithm was

(8) $$(4n+D\left(P_{n}\right)+D\left(G_{0}\right))R$$

where $D(P_{n})$ is the full depth of $P_{n}$, and $D(G_{0})$ is the full depth of $G_{0}$. Specifically, $D(P_{n})$ and $D(G_{0})$ were (84, 168) and (16, 32) in SAND-64 and SAND-128.

In our study, we undertook a comparison of the implementation of our quantum algorithm with other lightweight quantum cryptography implementations, as shown in Table 8. For certain studies, which are listed in Table 8, we performed a recalculation by implementing the decomposition of the Toffoli gate. Additionally, the depth-times-width value, which is denoted as D·W in the table, was computed. This value was determined by multiplying the T-gate depth (T-depth) by the number of qubits (width). The comparison revealed that SAND-64 exhibited the lowest depth-times-width value (154, 560) compared to the other lightweight ciphers. This indicated that the quantum circuit calculation cost associated with the SAND cipher algorithm was relatively low.

The number of qubits required in this brute-force attack was $Q_{b}+4n+1$, where $Q_{b}$ is the number of qubits required for the quantum implementation of SAND (the original Grover-based brute-force attack circuit requires $rQ_{b}+1$ qubits). There were (937,661) qubits required in SAND-64 and SAND-128 for the original attack framework, while fewer (363,587) qubits were required in SAND-64 and SAND-128 for a generalized brute-force attack.

Many works have assumed that T gates constitute the main cost ([15,16,17,25]), and T gates are exceptionally expensive for surface code [40]. It was assumed that the quantum implementation cost of Clifford gates was negligible in comparison with that of T gates in the dimension of $2^{k/2}$.

In κ iterations, the cost of implementing $U_{w}$ was considered. The consumption of the diffusion operator and the g−database unitary and state distinctions were ignored, and two SA instances are required. For this part of the calculation, the data in Table 8 could be used. The required number of T gates was

(9) $$\left\lceil \frac{\pi }{4}2^{64}\right\rceil \cdot 2\cdot \Delta_{T}$$

The required full depth was

(10) $$\left\lceil \frac{\pi }{4}2^{64}\right\rceil \cdot 2\cdot \Delta_{Full}$$

where $\left(\Delta_{T},\Delta_{Full}\right)$ in SAND-64 was (16128,10944), while the value in SAND-128 was (36288,24624). The gates required for SAND-64 were $\left\lceil \frac{\pi }{4}2^{64}\right\rceil \cdot 2\cdot 16128\approx 2^{78.628}$, while the gates required for SAND-128 were $\left\lceil \frac{\pi }{4}2^{64}\right\rceil \cdot 2\cdot 36288\approx 2^{79.798}$. The full depth required for SAND-64 was $\left\lceil \frac{\pi }{4}2^{64}\right\rceil \cdot 2\cdot 10944\approx 2^{78.069}$, while the full depth required for SAND-128 was $\left\lceil \frac{\pi }{4}2^{64}\right\rceil \cdot 2\cdot 24624\approx 2^{79.239}$.

The NIST defined the post-quantum security level according to the relative resource cost of quantum attacks that violate the security of AES-128, AES-192, and AES-256 [5]. The costs for security levels I, III, and V are estimated, respectively, as $2^{157}$, $2^{221}$, and $2^{285}$ computational resources (computational resources are calculated using gates multiplied by depth). The cost of the Grover-based brute-force attack on SAND-64 was $2^{78.628}\times 2^{78.069}\approx 2^{156.697}$, and that for SAND-128 was $2^{79.798}\times 2^{79.239}\approx 2^{159.037}$. The SAND-128 algorithm successfully achieved the NIST security level I ($2^{159.037}>2^{157}$). In contrast, the SAND-64 algorithm fell short of meeting the requirements of security level I ($2^{156.919}<2^{157}$).

The specific statistics are shown in Table 9.

## 6. Conclusions

This study provides a detailed quantum implementation of a lightweight block cipher SAND algorithm and describes the application of the Grover algorithm to the SAND algorithm under a generalized brute-force attack. Compared with other lightweight cryptographic quantum circuit implementations, the proposed quantum circuit implementation of SAND has relatively low quantum resource consumption for the depth-times-width metric. Regarding the security levels specified by the NIST, the SAND-128 algorithm achieved the NIST security level I, while the SAND-64 algorithm fell short of meeting the requirements of security level I. Our future work will concentrate on developing more optimized quantum circuit designs to minimize quantum resource usage for the SAND algorithm. 

## Figures and Tables

**Table 1:** Symbol table.

Symbol	Explain
$x=(x_{n-1},x_{n-2},\ldots ,x_{0})$	The variable *x* has a length of *n* and nmod4≡0.
x ‖ y	Link variable *x* and *y*.
x{i}	The *i*-th row element of the variable x(0≤i<4), i.e., $x\left\{3\right\}=\left(x_{n-1},\ldots ,x_{7},x_{3}\right),x\left\{2\right\}=\left(x_{n-2},\ldots ,x_{6},x_{2}\right),$ $x\left\{1\right\}=\left(x_{n-3},\ldots ,x_{5},x_{1}\right),x\left\{0\right\}=\left(x_{n-4},\ldots ,x_{4},x_{0}\right)$.
x[j]	The *j*-th nibble of variable x(0≤j<n/4), i.e., $x\left[\frac{n}{4}-1\right]=\left(x_{n-1},x_{n-2},x_{n-3},x_{n-4}\right),\ldots ,$ $x\left[1\right]=\left(x_{7},x_{6},x_{5},x_{4}\right),x\left[0\right]=\left(x_{3},x_{2},x_{1},x_{0}\right)$.
x≪s	Shift movement operation; *x* is shifted by *s* bits to the left.
x⋘t	Rotation operation; *x* is rotated by *t* bits to the left.
$x{⋘}_{n/4}t$	Row element x{i} of the variable *x* rotates *t* bits to the left, i.e., $x{⋘}_{n/4}t=\left(x\left\{3\right\}⋘t\right)\left|\right|$(x{2}⋘t)||(x{1}⋘t)||(x{0}⋘t).
x⊙y	And operation of variables *x* and *y*.
x⊕y	XOR operation of variables *x* and *y*.

**Table 2:** Relevant parameters for SAND-64 and SAND-128 .

SAND Version	Block Size 2n	Branch Size *n*	Key Size *k*	Rounds *R*
SAND-64	64	32	128	48
SAND-128	128	64	128	54

**Table 3:** $p_{8}$ for SAND-64.

j	0	1	2	3	4	5	6	7
$p_{8}\left(j\right)$	7	4	1	6	3	0	5	2

**Table 4:** $p_{16}$ for SAND-128.

j	0	1	2	3	4	5	6	7	8	9	10	11	12	13	14	15
$p_{16}\left(j\right)$	14	15	8	9	2	3	12	13	6	7	0	1	10	11	4	5

**Table 5:** Implementation of $p_{8}$ and $p_{16}$.

Type	Sequence	SWAP	Sequence	SWAP	Sequence	SWAP
$p_{8}$	1	$x_{0},x_{7}$	4	$x_{0},x_{4}$	7	$x_{0},x_{5}$
	2	$x_{0},x_{2}$	5	$x_{0},x_{3}$		
	3	$x_{0},x_{1}$	6	$x_{0},x_{6}$		
$p_{16}$	1	$x_{0},x_{14}$	6	$x_{0},x_{12}$	11	$x_{1},x_{9}$
	2	$x_{0},x_{4}$	7	$x_{0},x_{10}$	12	$x_{1},x_{7}$
	3	$x_{0},x_{2}$	8	$x_{1},x_{15}$	13	$x_{1},x_{13}$
	4	$x_{0},x_{8}$	9	$x_{1},x_{5}$	14	$x_{1},x_{11}$
	5	$x_{0},x_{6}$	10	$x_{1},x_{3}$		

**Table 6:** The consumption of components of the SAND algorithm.

Stages	Component	Number	CNOT	H	X	T	T-Depth
Round function	C1	2	(84, 180) ^3^	-	-	-	-
	$G_{1}$	2	(96, 192)	(32, 64)	-	(112, 224)	(4, 8)
	$G_{0}$	1	(96, 192)	(32, 64)	-	(112, 224)	(6, 12)
	$P_{n}$	1	(84, 168)	-	-	-	-
	CNOTS ^1^	4	(32, 64)	-	-	-	-
	INIT	1	-	-	(16, 32)	-	-
Key schedule	$A_{n/4}$	6	(48, 48)	-	-	-	-
	RC ^2^	1	-	-	(94, 104)	-	-
	CNOTS ^1^	N/A	(38, 70)	-	-	-	-

^1^ The CNOT gates establish connections between different components. ^2^ The consumption of the round constant for all rounds. ^3^ The data in the table represent the quantum resource consumption for SAND-64 and SAND-128.

**Table 7:** The consumption of a round of the SAND algorithm.

Cipher	Round Function				Key Schedule			
	**#Clifford**	**#T**	**#T-Depth**	**#Qubit**	**#Clifford**	**#T**	**#T-Depth**	**#Qubit**
SAND-64	780	336	14	96	326 + $\eta_{1}$ ^1^	N/A	N/A	138
SAND-128	1584	672	28	192	358 + $\eta_{2}$ ^1^	N/A	N/A	138

^1^$\left(\eta_{1},\eta_{2}\right)$ is the Clifford gate in SAND-64 and SAND-128 for obtaining the round constant for every round function.

**Table 8:** Quantum consumption of the SAND algorithm compared with that of other lightweight algorithms.

Cipher	Version	#Clifford	#T	#Full-Depth	#T-Depth	#Qubit	D·W
SAND	SAND-64	57,600	16,128	10,944	672	234	154,560
	SAND-128	110,484	36,288	24,624	1512	330	492,912
CHACHA [25]	CHACHA-12	141,344	90,272	27,439	11,904	1024	12,189,696
	CHACHA-20	228,640	145,824	45,359	19,840	1024	20,316,160
DEFAULT [38]	DEFAULT2022	75,371	57,344	2291	1024	256	262,144
	DEFAULT2021	89,975	62,720	2497	1120	640	716,800
SM4 (Stand-alone) [24]		378,204	49,152	Not reported	455	1464	666,120
CHAM-64/128 (FSE) [27]		36,920	16,240	17,031	9280	195	1,809,600
SPECK-64/128 [39]		39,664	22,631	13,365	6588	194	1,278,072
LowMC-L1/Regular [39]		500,674	4200	4708	240	3200	768,000

**Table 9:** Cost estimates of brute-force attack using the Grover algorithm for SAND.

Version	Gates	Depth	Cost	NIST Security
SAND-64	78.628	78.069	156.697	Not achieved
SAND-128	79.798	79.239	159.037	level I

These values are represented on the log scale.

**Figure A1:**
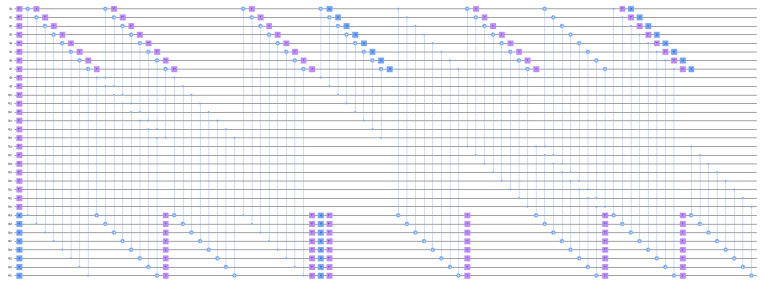
Implementation of quantum circuits of $G_{0}$ in SAND-64.

**Figure A2:**
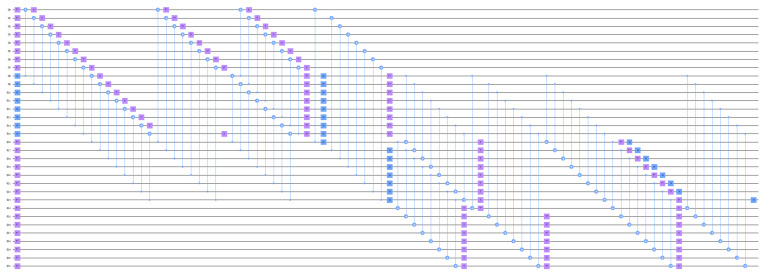
Implementation of quantum circuits of $G_{1}$ in SAND-64.

## Data Availability

The data are contained within the article.

## Abbreviations

The following abbreviations are used in this manuscript:

DCC	Designs, Codes, and Cryptography
NIST	National Institute of Standards and Technology
PQC	Post-Quantum Cryptography
AES	Advanced Encryption Standard
S-box	Substitution Box
AND-RX	AND-Recursive exchange
SAND	S-box-based and AND-RX-based structures

## References

[1] Mosca M. (2018). Cybersecurity in an era with quantum computers: Will we be ready?. IEEE Secur. Priv..

[2] Schrottenloher A. (2021). Quantum Algorithms for Cryptanalysis and Quantum-Safe Symmetric Cryptography. Ph.D. Thesis.

[3] Shor P.W. (1999). Polynomial-time algorithms for prime factorization and discrete logarithms on a quantum computer. SIAM Rev..

[4] Grover L.K. A fast quantum mechanical algorithm for database search. Proceedings of the Twenty-Eighth Annual ACM Symposium on Theory of Computing.

[5] NIST Call for Additional Digital Signature Schemes for the Post-Quantum Cryptography Standardization Process 2022. https://csrc.nist.gov/csrc/media/Projects/pqc-dig-sig/documents/call-for-proposals-dig-sig-sept-2022.pdf.

[6] Caleffi M., Chandra D., Cuomo D., Hassanpour S., Cacciapuoti A.S. (2020). The rise of the quantum internet. Computer.

[7] Lloyd S., Shapiro J.H., Wong F.N., Kumar P., Shahriar S.M., Yuen H.P. (2004). Infrastructure for the quantum Internet. ACM SIGCOMM Comput. Commun. Rev..

[8] Thapliyal H., Muñoz-Coreas E., Khalus V. (2021). Quantum circuit designs of carry lookahead adder optimized for T-count T-depth and qubits. Sustain. Comput. Inform. Syst..

[9] Park J.J., Baek K., Kim M., Nha H., Kim J., Bang J. (2023). T-depth-optimized quantum search with quantum data-access machine. Quantum Sci. Technol..

[10] Larasati H.T., Putranto D.S.C., Wardhani R.W., Park J., Kim H. (2023). Depth Optimization of FLT-Based Quantum Inversion Circuit. IEEE Access.

[11] López L.O., Orts F., Ortega G., González-Ruiz V., Garzón E.M. (2023). Fault-tolerant quantum algorithm for dual-threshold image segmentation. J. Supercomput..

[12] Qin D., Chen Y., Li Y. (2023). Error statistics and scalability of quantum error mitigation formulas. NPJ Quantum Inf..

[13] DeCross M., Chertkov E., Kohagen M., Foss-Feig M. (2023). Qubit-reuse compilation with mid-circuit measurement and reset. Phys. Rev. X.

[14] Osvik D.A., Bos J.W., Stefan D., Canright D. (2010). Fast software AES encryption. Proceedings of the International Workshop on Fast Software Encryption.

[15] Grassl M., Langenberg B., Roetteler M., Steinwandt R. (2016). Applying Grover’s algorithm to AES: Quantum resource estimates. Proceedings of the International Workshop on Post-Quantum Cryptography.

[16] Almazrooie M., Samsudin A., Abdullah R., Mutter K.N. (2018). Quantum reversible circuit of AES-128. Quantum Inf. Process..

[17] Jaques S., Naehrig M., Roetteler M., Virdia F. (2020). Implementing Grover oracles for quantum key search on AES and LowMC. Proceedings of the Advances in Cryptology–EUROCRYPT 2020: 39th Annual International Conference on the Theory and Applications of Cryptographic Techniques.

[18] Zou J., Wei Z., Sun S., Liu X., Wu W. (2020). Quantum circuit implementations of AES with fewer qubits. Proceedings of the International Conference on the Theory and Application of Cryptology and Information Security.

[19] Huang Z., Sun S. (2022). Synthesizing quantum circuits of AES with lower t-depth and less qubits. Proceedings of the International Conference on the Theory and Application of Cryptology and Information Security.

[20] Luo Q.B., Yang G.W., Li X.Y., Li Q. (2022). Quantum reversible circuits for multiplicative inverse. EPJ Quantum Technol..

[21] Rajesh S., Paul V., Menon V.G., Khosravi M.R. (2019). A secure and efficient lightweight symmetric encryption scheme for transfer of text files between embedded IoT devices. Symmetry.

[22] Singh S., Sharma P.K., Moon S.Y., Park J.H. (2017). Advanced lightweight encryption algorithms for IoT devices: Survey, challenges and solutions. J. Ambient. Intell. Humaniz. Comput..

[23] LIN D., XIANG Z., ZHANG R., ZHANG S., ZENG X. (2021). Quantum implementation of SM4. J. Cryptologic Res..

[24] Zou J., Li L., Wei Z., Luo Y., Liu Q., Wu W. (2022). New quantum circuit implementations of SM4 and SM3. Quantum Inf. Process..

[25] Bathe B., Anand R., Dutta S. (2021). Evaluation of Grover’s algorithm toward quantum cryptanalysis on ChaCha. Quantum Inf. Process..

[26] Jang K., Choi S., Kwon H., Kim H., Park J., Seo H. (2020). Grover on Korean block ciphers. Appl. Sci..

[27] Yang Y., Jang K., Baksi A., Seo H. (2023). Optimized implementation and analysis of cham in quantum computing. Appl. Sci..

[28] Chen S., Fan Y., Sun L., Fu Y., Zhou H., Li Y., Wang M., Wang W., Guo C. (2022). SAND: An AND-RX Feistel lightweight block cipher supporting S-box-based security evaluations. Des. Codes Cryptogr..

[29] Pan S.J., Wan L.C., Liu H.L., Wang Q.L., Qin S.J., Wen Q.Y., Gao F. (2020). Improved quantum algorithm for A-optimal projection. Phys. Rev. A.

[30] Foss-Feig M., Hayes D., Dreiling J.M., Figgatt C., Gaebler J.P., Moses S.A., Pino J.M., Potter A.C. (2021). Holographic quantum algorithms for simulating correlated spin systems. Phys. Rev. Res..

[31] Bonnetain X., Hosoyamada A., Naya-Plasencia M., Sasaki Y., Schrottenloher A. (2019). Quantum attacks without superposition queries: The offline Simon’s algorithm. Proceedings of the Advances in Cryptology–ASIACRYPT 2019: 25th International Conference on the Theory and Application of Cryptology and Information Security.

[32] Buhrman H., Cleve R., Watrous J., De Wolf R. (2001). Quantum fingerprinting. Phys. Rev. Lett..

[33] Lu D., Li Z., Yu J., Han Z. (2022). A verifiable arbitrated quantum signature scheme based on controlled quantum teleportation. Entropy.

[34] Chen F.L., Wang Z.H., Hu Y.M. (2019). A new quantum blind signature scheme with BB84-state. Entropy.

[35] Carstens T.V., Ebrahimi E., Tabia G.N., Unruh D. (2021). Relationships between quantum IND-CPA notions. Proceedings of the Theory of Cryptography: 19th International Conference, TCC 2021.

[36] Selinger P. (2013). Quantum circuits of T-depth one. Phys. Rev. A.

[37] Amy M., Maslov D., Mosca M., Roetteler M. (2013). A meet-in-the-middle algorithm for fast synthesis of depth-optimal quantum circuits. IEEE Trans.-Comput.-Aided Des. Integr. Circuits Syst..

[38] Jang K., Baksi A., Breier J., Seo H., Chattopadhyay A. (2023). Quantum implementation and analysis of default. Cryptogr. Commun..

[39] Jang K., Baksi A., Kim H., Seo H., Chattopadhyay A. (2022). Improved quantum analysis of SPECK and lowmc. Proceedings of the International Conference on Cryptology in India.

[40] Fowler A.G., Mariantoni M., Martinis J.M., Cleland A.N. (2012). Surface codes: Towards practical large-scale quantum computation. Phys. Rev. A.

